# The Habitat Fragmentation and Suitability Evaluation of Mrs Hume’s Pheasant *Syrmaticus humiae* in Northwestern Guangxi, China

**DOI:** 10.3390/biology14101345

**Published:** 2025-10-01

**Authors:** Baodong Yuan, Ying Li, Zhicheng Yao

**Affiliations:** 1 College of Agriculture and Biology, Liaocheng University, Liaocheng 252000, China; 2 Zibo Forestry Protection and Development Center, Zibo 255000, China

**Keywords:** Mrs Hume’s pheasant, habitat, landscape pattern, fragmentation, Jinzhongshan

## Abstract

**Simple Summary:**

The Jinzhongshan region covers 38,716.6 hm^2^, including 1708 patches and 11 landscape types. Although the area of farmland and village only occupies 10%, their number and density have led Jinzhongshan habitats to fragment. The degree of connection of suitable habitat was found to be relatively low, and seven landscape indices were below 0.5, which implied that the extent of habitat fragmentation in Jinzhongshan for Mrs Hume’s pheasant is very high. The fragmentation index of Jinzhongshan Nature Reserve is 0.9887, landscape connectivity is 1.861, and AWS index is 425.3024. The broad-leaved forest, considered a matrix in the Jinzhongshan area, was the dominant landscape type controlling structure, function, and dynamic changes.

**Abstract:**

The habitat landscape pattern of Mrs Hume’s pheasant in Jinzhongshan, northwestern Guangxi, was studied using field survey data and the LANDSAT satellite images by the ArcGIS 10.8 and Fragstats 3.3 software. The results showed that the Jinzhongshan region covers 38,716.6 hm^2^, including 1708 patches and 11 landscape types. Although the area of farmland and village only occupies 10%, their number and density have led Jinzhongshan habitats to fragment. The degree of connection of suitable habitat was found to be relatively low, and seven landscape indices were below 0.5, which implied that the extent of habitat fragmentation in Jinzhongshan for Mrs Hume’s Pheasant is very high. The fragmentation index of Jinzhongshan Nature Reserve is 0.9887, landscape connectivity is 1.861, and AWS index is 425.3024. The broad-leaved forest, considered a matrix in the Jinzhongshan area, was the dominant landscape type controlling structure, function, and dynamic changes. The total suitable habitat of Mrs Hume’s pheasant (*Syrmaticus humiae*) was determined to be 29,552.3 hm^2^, accounting for 76.3% of the total study area; the suitable habitat of Mrs Hume’s pheasant in Jinzhongshan Nature Reserve was determined to be 16,990.1 hm^2^, accounting for 81.14% of the protected area. It is absolutely necessary and urgent to strengthen the management and protection of Mrs Hume’s pheasant’s habitat to control its fragmentation. Therefore, we have provided some useful advice, such as habitat restoration and corridor reconstruction, which are beneficial to the conservation of Mrs Hume’s pheasant in this sensitive region.

## 1. Introduction

The properties of the landscape, such as patch size, edge quantity, distance between habitats, and connectivity between habitat patches, have a direct impact on the flora and fauna of the region [1]. The analysis of landscape patterns can use specific landscape indices to describe abstract spatial patterns and deduce the mechanisms and causes of landscape patterns from seemingly disordered landscape elements [2,3,4]. The factor that has a relatively large impact on the natural landscape pattern is human interference [5,6]. Due to human activities changing the landscape’s spatial structure, many species are usually confined to small, isolated, and degraded habitat patches, which affects the survival of animals. The mechanism of landscape fragmentation or animal extinction caused by landscape patterns has become a hot issue in conservation biology [7,8,9,10]. It is considered one of the main reasons for the endangerment and extinction of many species and the decline of biodiversity [11]. Landscape pattern analysis, as the basic research content of landscape ecology, can quantitatively analyze the spatial distribution characteristics of landscape components and is the basis for further research on landscape functions and dynamics. Conducting these studies will help solve some important theories and key technical issues in the effective in situ conservation of biodiversity [12].

Different species respond differently to habitat fragmentation. The Worm-eating Warbler (*Helmitheros vermivorus*), Middle Spotted Woodpecker (*Dendrocopos medius*), and Ovenbird (*Seiurus aurocapillus*) in North America—all area-sensitive species that mostly inhabit forest interiors—appear more frequently in continuous landscapes, but their densities decrease in fragmented landscapes [13]. In contrast, the Northern Cardinal (*Cardinalis cardinalis*) and Indigo Bunting (*Passerina cyanea*), which nest in edge habitats, secondary forests, or urbanized areas, exhibit higher densities in fragmented rather than in continuous landscapes [14,15]. Research on Amazon rainforest fragmentation further shows that species richness is positively correlated with patch size [16,17,18]. Notably, responses to fragmentation vary substantially even within closely related taxa [19]. For species of the genus Syrmaticus (to which this study’s focal species, Mrs. Hume’s pheasant, belongs) and their relatives—groups that have been intensively studied using molecular genetics, inbreeding analysis, and isolation effect assessments—habitat fragmentation often exerts taxon-specific pressure. Molecular genetic studies of Syrmaticus species (e.g., *Syrmaticus humiae* and *Syrmaticus reevesii*) have revealed that population genetic diversity is closely linked to habitat connectivity: fragmented habitats reduce gene flow among subpopulations, leading to increased genetic differentiation [19]. Recent inbreeding analysis of Syrmaticus populations further confirms that fragmented habitats elevate inbreeding levels, with inbreeding depression (e.g., reduced reproductive success and survival rates) becoming more pronounced in highly isolated patches [20]. For insular Syrmaticus populations, such as those on Taiwan Island, geographic isolation (exacerbated by anthropogenic fragmentation) has driven significant genetic divergence from mainland conspecifics, highlighting the long-term evolutionary impacts of habitat disconnection [21,22]. In tropical regions, 65% of natural habitats have disappeared [23]. In temperate regions, the original natural habitats no longer exist [24]. In China, habitat fragmentation reduces the quality and area of habitats, having an important impact on the survival of animals [25,26,27]. Habitat fragmentation, or fragmentation, has an important impact on the breeding success rate, population viability, dispersal behavior, habitat selection, genetic diversity of birds, and the resulting ecological effects (such as edge effects and isolation effects) [28,29,30,31].

Among avian species, edge responses also vary: Steller’s Jay (*Cyanocitta stelleri*) and Swainson’s Thrush (*Catharus ustulatus*) have higher densities in edge areas, while the Brown Creeper (*Certhia americana*), Pacific-slope Flycatcher (*Empidonax difficilis*), and Varied Thrush (*Ixoreus naevius*) rarely occur in edge habitats [20,21,22]. For other taxa, responses to fragmentation also diverge: some species are highly sensitive to area loss in fragmented forest habitats, while the species richness of taxa like frogs and butterflies remains stable or even increases after forest patch isolation [19,32,33,34]. However, it is important to note that amphibians (including frog species) often face hidden risks in fragmented landscapes: recent studies have documented inbreeding depression in amphibian populations in fragmented urban or agricultural areas, where reduced connectivity limits gene flow and increases the frequency of deleterious alleles [34,35,36,37]. Urbanization, in particular, intensifies such effects by creating permanent barriers to dispersal, further exacerbating genetic degradation in amphibian populations [38,39,40,41,42,43].

The Mrs. Hume’s pheasant (*Syrmaticus humiae*) is listed as a globally near-threatened species by the IUCN Red List [27] and is particularly vulnerable to habitat fragmentation due to its weak migration and dispersal abilities—traits that limit its capacity to colonize new habitats or connect isolated subpopulations [30,37]. Geographically, the species has a disjunct distribution across Southeast Asia and southern China, with core populations in Guangxi, Yunnan, and Guizhou provinces of China, as well as in northern Myanmar and northeastern India (GBIF, 2024. *Syrmaticus humiae* (Hume, 1881). Available online: https://www.gbif.org/species/2473479, accessed on 29 September 2025). Based on GBIF occurrence data and regional surveys, its global population size is estimated to be 10,000–19,999 mature individuals, with a declining trend: in China, local abundances range from 0.1 to 0.5 individuals per km^2^ in moderately disturbed habitats to 1.0–2.0 individuals per km^2^ in well-protected forest patches (e.g., in Jinzhongshan Nature Reserve, Guangxi) [27]. Like other gallinaceous birds, *S. humiae* faces severe threats from human activities and climate change—two primary drivers of galliform population decline and extinction globally [27]. Human-induced habitat loss (e.g., deforestation for agriculture, logging) and fragmentation have reduced its suitable habitat by over 30% in the past three decades [44,45]. Climate change further exacerbates these threats by shifting the species’ climatic niche, leading to mismatches between its current distribution and suitable environmental conditions (e.g., increased drought frequency reducing understory vegetation cover, a key resource for foraging and nesting). A case in point is another Syrmaticus species, Reeves’s Pheasant (*Syrmaticus reevesii*), which has experienced a more severe population collapse: its rapid decline due to habitat destruction and overhunting led to its elevation to China’s National First-Class Protected Animal status [27,45]. However, for many Syrmaticus species (including S. humiae), identifying the precise drivers of population decline and implementing effective conservation measures remain challenging, largely due to the lack of long-term, high-spatiotemporal-resolution data on population dynamics, habitat use, and threat impacts. To address this gap, the present study identifies potential ecological corridors for *S. humiae* in Jinzhongshan using the Minimum Cumulative Resistance (MCR) model—a widely applied tool for prioritizing connectivity conservation [20]. This approach provides actionable insights for mitigating fragmentation impacts and safeguarding the species’ long-term survival. Some scholars have reported on the habitat use and population distribution of the Mrs Hume’s pheasant in Thailand [31,32,33,34] and the characteristics and use of its spring habitat [35]. The summer habitat selection of the re-introduced Mrs Hume’s pheasant in Cenwanglaoshan was also reported [37,38]. With the intensification of human interference, habitat loss and fragmentation have become some of the most significant threats to biodiversity [39,40]. Analyzing habitat fragmentation and conducting adaptability evaluations can provide a basis for the protection of endangered species [44,45,46,47]. In terms of the habitat evaluation of the Mrs Hume’s Pheasant, only one recent study on the suitability of its macro-habitat scale in the Nanhua section of Ailao Mountain, Yunnan, has been conducted [48,49]. The research results show that the suitable habitats have been fragmented.

Jinzhongshan, Guangxi, is a key distribution area of the Mrs Hume’s pheasant in China, according to previous studies of Mrs. Hume’s pheasant by Jiang et al. (2012) and Yuan et al. (2024). Twenty (20) ecological variables were considered as potential factors affecting the habitat selection of Mrs. Hume’s pheasant [27,45], and the main habitat types of the Mrs Hume’s pheasant are Pinus yunnanensis var. tenuifolia forests, coniferous broad-leaved mixed forests, and evergreen–deciduous broad-leaved mixed forests, but it can also be active in tung oil forests and artificial Chinese fir forests [45]. There are currently no reports on relevant habitat fragmentation and habitat evaluation. Therefore, analyzing the landscape pattern and fragmentation degree of the habitat of the Mrs Hume’s pheasant and evaluating the habitat suitability to explore the spatial utilization pattern of the habitat of the Mrs Hume’s pheasant in fragmented habitats are highly necessary [46,47,48]. At the same time, understanding the current situation of habitat interference and damage of the Mrs Hume’s pheasant provides a theoretical basis for corridor restoration and connectivity improvement. The research results are of great significance for the design and management planning of nature reserves.

## 2. Research Methods

### 2.1. Geographical Location

**Figure 1:**
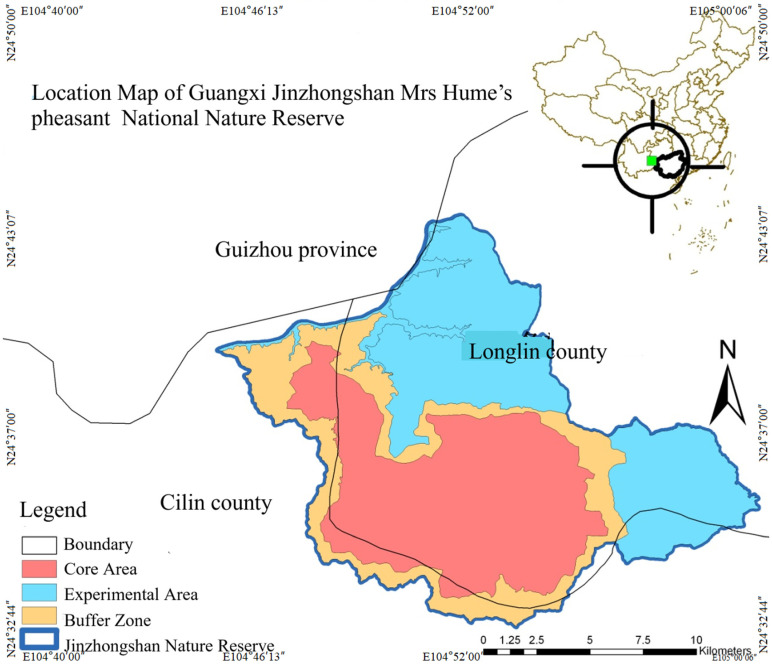
Location map of Mrs Hume’s pheasant in Guangxi Jinzhongshan National Nature Reserve.

Guangxi Jinzhongshan Nature Reserve is located in the northwest of the Guangxi Zhuang Autonomous Region, with geographical coordinates ranging from 104°46′13″ to 105°00′06″ east longitude and from 24°32′06″ to 24°43′07″ north latitude. The total area of the Nature Reserve is 20,924.4 hm^2^, including a core area of 8404.3 hm^2^, a buffer area of 4262.4 hm^2^, and an experimental area of 8227.7 hm^2^ (Figure 1). The climate here is often regulated by the oceanic air currents from the Beibu Gulf and the Bay of Bengal, showing obvious oceanic characteristics. The climate is mild throughout the year, with an average annual temperature of 17.1 °C. The average temperature in July is 23.4 °C, and the average temperature in January is 8.3 °C. The annual temperature difference is relatively small, while the daily temperature difference is relatively large. It is warm in winter and spring, cool in summer, and has a short frost period [14,44,45].

The area enjoys abundant sunlight, with an annual sunshine duration of 1569.3 h and an annual total solar radiation of 4329 MJ/m^2^. It is one of the regions in Guangxi with relatively rich solar energy resources. The annual rainfall is 1262.8 mm, making it one of the regions in Guangxi with relatively low precipitation. Jinzhongshan is rich in plant species. There are 1487 species (including varieties) of wild vascular plants belonging to 725 genera in 193 families. There are three species of first-class protected plants, namely *Cycas guizhouensis*, *C. longliensis,* and *Bretschneidora sinensis*. There are 389 species of terrestrial vertebrate wild animals, including 20 species of amphibians, 40 species of reptiles, 274 species of birds, and 55 species of mammals. Currently, there are 52 species of national key protected wild animals distributed here. Among them, there are 5 species of national first-class key protected wild animals, including the Mrs Hume’s pheasant, *Aquila chrysaetos*, *Python molurus*, *Neofelis nebulosa*, and *Moschus berezovskii*, and 47 species of national second-class key protected wild animals, such as *Hoplobatrachus rugulosus, Palea steindachneri, Aix galericulata, Aviceda leuphotes, Macaca mulatta*, and *Manis pentadactyla*.

Eight Mrs. Hume’s pheasant (two females and six males; 4% of the total bird population) were captured with the slingshot trap method (Table 1). All the eight birds were equipped with radio transmitter sets (RI-2D) with the radio frequency between 150.000 and 150.700 MHz, then released to the wild quickly [44]. The weight of the radio transmitter set was 17 g, approximately 2% of the body mass of the target birds. Monitoring was accomplished by three-element Yagi antennae to track signals and then identify bird position and the home region. The software program Location of a Signal, version 4.0 (Ecological Software Solutions LLC, Panama City Beach, FL, USA), was used to generate an estimated location and an error ellipse for radio-marked wild Mrs. Hume’s pheasant. During the study period, signals were received and recorded from 3 or 4 points from 6:00 to 18:00 h every day.

Radio-tracking methods were used to determine the used and available sites of all eight birds. In addition, habitats used by Mrs. Hume’s pheasant were located based on the activity evidence of its feces, plumes, sand or mud bath sites, individuals, etc. Using a portable GPS, every located site was geographically identified and parameters of latitude, longitude, and altitude were recorded. A sampling quadrat of 10 m × 10 m (100 m^2^) was subsequently marked to establish the used area at a particular site, and the habitat characteristics were measured. During the study period, 111 quadrats (48 in 2012 and 63 in 2013) were laid and data collected from all sites. A similarly marked site, located 50 m away from the used site, was established for the purpose of an unused site as control quadrat. Though many ecological factors would exert influences on animal habitat selection and use, we mainly studied the important factors for summer habitat use. In each located quadrat, necessary parametric measurements were recorded. Definition and measurement of 20 ecological variables, as shown in Table 2, were based on the framework of Zhou et al. (2010) and Yuan et al. (2024) [45,48]. Most of the ecological variables were determined in the 10 m × 10 m plots as continuous data and the latter lumped into categories; however, the leaf litter coverage, the height of herb species, the density of herbs, and the food abundance were collected in five (5) random 1.0 m × 1.0 m plots which were settled in the 10 m × 10 m plots as continuous data and the latter lumped into categories. Classification of ecological factor measures is detailed below.

### 2.2. Landscape Pattern

Based on the field surveys in the study area from 2009 to 2012, the vector maps of sub-compartments, the sub-compartment database from the second-class forest resource survey in the Nature Reserve in 2009, and the ETM + image of Jinzhongshan in 2010 as the satellite image, the exact months of image capture were April, August, and December 2012 to leverage seasonal vegetation indices (e.g., NDVI, EVI); interpretation was carried out using the ERDAS IMAGINE 9.0 remote-sensing software. Vectorization was performed on the 2009 LANDSAT satellite remote-sensing TM map (with a resolution of 30 m) using the ArcGIS 10.8 software. Trained on region-of-interest (ROI) samples from field surveys, the landscape types in the study area were divided into 11 types: evergreen broad-leaved forest, deciduous broad-leaved forest, evergreen–deciduous broad-leaved mixed forest, masson pine forest, Chinese fir forest, Pinus yunnanensis var. tenuifolia forest, tung oil (*Vernicia fordii*) forest, economic forest, water area, village, and farmland. Landscape indices can quantitatively describe the landscape pattern, establish the connection between landscape structure and processes or phenomena, and better understand and interpret landscape functions. Landscape analysis, as the basic research content of landscape ecology, can quantitatively analyze the spatial distribution characteristics of landscape components and is the basis for further research on landscape functions and dynamics. The relevant landscape indices were calculated using the landscape structure analysis software Fragstats 3.3 (raster version). The Cell size was selected as 5 m × 5 m, and the Cell assignment type was selected as CELL_CENTER. Fragstats can calculate 277 indicators at the patch, type, and landscape levels (for the calculation formulas and meanings of the above indices, refer to the software instruction package of Fragstats 3.3 (raster version) and the literature [45]). Among them, there are 22 patch-level indices, 123 patch-type-level indices, and 132 landscape-level indices, but these indicators are highly correlated (Table 3). Sixteen landscape indices commonly used to analyze landscape changes were selected. The fragmentation degree of each landscape was analyzed through the average patch area, total perimeter, proportion of the largest patch in the landscape area, patch density of the entire landscape, number of patches, patch density of the landscape components, perimeter–area fractal dimension, connectivity, and landscape shape index. Finally, the fragmentation degree of the study area was calculated using the fragmentation index (F) formula. The Shannon–Wiener diversity index, Simpson diversity index, evenness index, and contagion index were used to analyze landscape diversity [46]. The landscape pattern distribution map was drawn using the ArcGIS 10.8 software, and the results of the Fragstats 3.3 operation were organized using Excel 2019.

Among them, the patch shape index represents the degree of difference between the patch shape and a circle. The minimum value of this index is 1. The closer the value is to 1, the more similar the patch shape is to a circle. The larger the value, the greater the difference between the patch shape and a circle, and the more irregular the shape.

(1) Patch shape index Di = = $\frac{P_{i}}{2\sqrt{\pi A}}$ (Pi: patch perimeter);
(2) Area-weighted shape index AWS = AWS = $\sum_{i=1}^{i}\frac{A_{i}D}{A}$ (Ai: area of patch i; D: patch shape index; A: total area of forest patches);
(3) Connectivity index Ii = $\log\sum_{j=1}^{n}\sqrt{A_{i}A_{j}}\frac{\sqrt{A_{i}A_{j}}}{{d_{ij}}^{2}}$ (j≠i).

In the formula, Ii is the landscape connectivity index between the i-th patch and all other patches (Ii ≥ 0, and the smaller Ii is, the smaller the connectivity between patches. When Ii = 0, it means that the patch is completely isolated from other patches.). Aj is the area of the j-th patch (km^2^), and dij is the shortest distance between the boundaries of the i-th and j-th patches (km).

(4) Patch fragmentation F = n/A or F = $\frac{1}{A}\sum_{i=1}^{n}d_{ij}A_{i}$, fragmentation index F = 1 − $\sum_{i=1}^{n}\frac{1}{D_{i}}\;(Ai/A)2$ (Ai/A) (n: number of patches; Ai: area of the i-th patch; A: total area of the study area; D: patch shape index).

### 2.3. Habitat Suitability Evaluation Method

Habitat suitability evaluation can explore and predict the potential distribution of species, assess habitat quality, predict ecological carrying capacity, and plan and construct nature reserves. It plays an important role in effectively analyzing the endangerment mechanisms of species and formulating corresponding conservation measures [43].

#### 2.3.1. Flow of Habitat Suitability Evaluation of the Mrs Hume’s Pheasant

(1) Analyze the habitat requirements of the Mrs Hume’s pheasant and clarify the limiting or dominant factors affecting the population distribution and behavior of the Mrs Hume’s pheasant.
(2) Collect and prepare corresponding geographical data according to the evaluation factors, establish the evaluation criteria for each influencing factor, and complete the spatial analysis and processing of the data with the help of GIS technology to conduct the suitability evaluation of each single factor.
(3) Conduct a superposition analysis of each single factor with the help of GIS technology according to the evaluation criteria for comprehensive habitat analysis and evaluation.
(4) Evaluate the habitat suitability of the Mrs Hume’s pheasant based on the results.

#### 2.3.2. Selection of Habitat Evaluation Model for the Mrs Hume’s Pheasant

Currently, there are many models used to evaluate the habitat quality of animals, such as the Ecological Niche Factor Analysis (ENFA) model, Least-Cost Model, Spatiotemporally Explicit Model (SEM), Habitat Suitability Index (HSI) model, MAXENT model, etc. The Ecological Niche Factor Analysis model is a multivariate analysis method based on GIS technology used to study the geographical distribution of species and evaluate habitat suitability. It predicts habitat suitability by comparing the optimal ecological niche parameters of specific species [38]. The Least-Cost Model is a general research model based on the relationship between animal migration paths and landscape characteristics, used to identify the corridors for the diffusion of wild animal populations. The migration path calculated by the Least-Cost Model has the minimum migration resistance and the shortest migration distance between suitable habitats [39,40]. The Spatiotemporally Explicit Model is based on the Household and Landscape Integration Model (HALIM), used to examine the impact of abiotic factors on the spatial distribution pattern of human activities (such as livestock and firewood collection), and the impact of human activities on the quantity and pattern of habitats [44,45,46]. The Habitat Suitability Index model, as a habitat evaluation method, is widely used. Since the HSI model can identify the evaluation factors that have a greater impact on habitat quality, it can also be used to simulate the habitat quality of species after environmental changes, which has important guiding significance for the protection of endangered species [44]. The MAXENT model is a model with relatively high accuracy and is widely used in the evaluation of wildlife habitats internationally [48], but currently, there are few domestic studies on animal habitat evaluation using this model. In addition, the research team developed a giant panda habitat evaluation system based on the theories of landscape ecology and spatial analysis using VB.NET and ArcEngine and proposed a method for extracting core patches and potential corridors based on raster data [44]. This evaluation system is convenient for users to achieve habitat evaluation and landscape pattern design of nature reserves and has a high degree of interactivity with users. Abroad, the habitat quality of the Bale Mountains Bushbuck (*Tragelaphus buxtoni*) in Ethiopia was analyzed using the FunConn model based on spatial expertise, which has good accuracy in evaluating habitat quality. The ecological niche of an animal’s resource requirements is a multi-dimensional space composed of multiple resources. According to Shelford’s law of tolerance, if any ecological factor is insufficient in quantity or quality, the species will decline or be unable to survive. The landscape connectivity model was selected to evaluate the habitat suitability. Based on this model, not only can the species–environment relationship be studied quantitatively and at multiple scales, the suitable habitats of species can be determined, and a species habitat suitability distribution map can be generated, but it can also be used in fields such as geographical distribution and biodiversity conservation [42].

The expression is as follows:

$$S_{j}\;=\;\prod_{i=1}^{n}S_{i}$$

where Sj represents the landscape connectivity level of different units, that is, the product of the importance values of each ecological factor; n represents the number of selected ecological factors, which is 4 here; Si represents the importance assignment of each ecological factor to the habitat selection of the Mrs Hume’s pheasant; and the value of Sj is between 0 and 1. Referring to the previous conclusions of the research on the habitat of the Mrs Hume’s pheasant [12,17,27], the vegetation type is the main factor affecting habitat selection, and the food factor is closely related to the vegetation type. The vegetation type shows a regular distribution with the increase in altitude, so geographical factors were not considered. When classifying habitat suitability, there is no fixed standard for determining the threshold for suitable or unsuitable habitats. A smaller threshold makes the area of suitable habitats larger and more statistically significant. According to the differences in Sj values, the potential habitats of the Mrs Hume’s pheasant (*Syrmaticus ellioti*) are divided into four grades. Among them, when the Sj value is greater than 0.667, it is the most suitable habitat; when the Sj value is greater than 0.333, it is a suitable habitat; when the Sj value is between 0 and 0.333, it is a marginally suitable habitat; and when the Sj value is 0, it is an unsuitable habitat (Table 4).

## 3. Results and Analysis

### 3.1. Area Characteristics of Landscape Components

The 2012 survey by the Guangxi Forestry Bureau indicated that the reserve hosted 200 individuals with a patchy distribution. The results indicated that the habitat characteristics were as follows: 200 m to 400 m distance to habitat edge, less than 200 m distance to water, over 400 m distance to human habitation, over 601 m to the road, over 60% tree cover, less than 40% shrub cover, less than 20% herb cover, over 60% leaf litter cover, over 10.1 m high trees, over 20 (individuals/per quadrat) tree density, over 2.1 m high shrubs, less than 10 (individuals/per quadrat) density of shrubs, over 0.5 m high herb species, less than 10 (individuals/per quadrat) density of herbs, over 30 cm diameter trees, and abundant food (Table 2) [44,45].

The proportion of a landscape type in the total landscape area represents the relative contribution of this landscape to the entire landscape. As can be seen from Table 3, the proportions of landscape types in the total landscape area, in descending order, are CKL > LKL > YT > NT > ML > SL > CLKL > SY > JJL > YNL > CZ. In the entire landscape, the area of evergreen broad-leaved forest accounts for the largest proportion of the total area, while the proportion of villages is the smallest. The areas of deciduous broad-leaved forest, evergreen broad-leaved forest, and evergreen–deciduous broad-leaved mixed forest account for 55.25% of the entire landscape. Broad-leaved forest is the matrix of the entire landscape, and coniferous forest, tung tree forest, economic forest, and other landscape components are mosaically distributed within it (Table 5).

The order of the average area of patches of each landscape component is SY > LKL > CLKL > CKL > YT > YNL > JJL > ML > SL > NT > CZ. In particular, the average areas of masson pine forest, Chinese fir forest, farmland, and villages are all smaller than the average area of patches in the entire landscape, indicating a high degree of fragmentation. The area proportion of Pinus yunnanensis var. tenuifolia forest is relatively small, but its average patch area is close to that of evergreen broad-leaved forest and tung tree forest, suggesting a relatively low degree of fragmentation. The water area is on the edge of the study area, accounting for a small proportion in the entire landscape and having little impact on other landscapes.

### 3.2. Perimeter of Landscape Components

The order of the perimeters of each landscape component is CKL > LKL > NT > YT > ML > SL > CLKL > JJL > SY > YNL > CZ. The general trend is slightly different from that of the landscape component area. In the study area, the evergreen broad-leaved forest has the highest proportion of the total perimeter, reaching 25.32%, followed by the deciduous broad-leaved forest, and the proportions of the rest are all less than 15%. The proportion of the perimeter of each landscape type to the total landscape perimeter is of great significance for the study of some species distributed at the boundaries. The evergreen broad-leaved forest and deciduous broad-leaved forest have large areas and can accommodate more boundary species.

### 3.3. Number of Landscape Component Patches

The number of patches of each landscape component in the study area is extremely unbalanced. Farmland has the largest number of patches, with 406 patches, accounting for 23.77% of the total number of patches. It is followed by evergreen broad-leaved forest, Chinese fir forest, masson pine forest, deciduous broad-leaved forest, and tung tree forest, and the proportions of the rest are all less than 10%. However, the water area, which has the largest average patch area, only has three patches. The number of farmland patches is much larger than that of evergreen broad-leaved forest and deciduous broad-leaved forest, but its total area is much smaller than the two. The proportion of the area of farmland in the total area is similar to that of masson pine forest, and the average patch areas are not very different, but the number of farmland patches is also much higher than that of masson pine forest patches, indicating that farmland has a large number of small-sized patches and a high degree of fragmentation.

The patch density PD1 of each landscape component (number of patches of the landscape component/area of the landscape component) directly reflects the degree of fragmentation of the landscape component. The village has the highest patch density, which is much higher than the average patch density of the entire landscape (total number of patches/total area), and has a greater impact on other landscapes. However, its area accounts for only 0.9% of the total area (PLAND), so it does not play a leading role in the fragmentation of the entire landscape. The patch density of the tung tree forest is similar to that of the economic forest, indicating that their degrees of fragmentation are similar. However, the area of the tung tree forest is much larger than that of the economic forest, so it has the greatest impact on the entire landscape.

The ratio of the number of patches of a type to the total landscape area represents the degree to which the landscape matrix is divided by the patches of this type, that is, the patch density PD2 (also known as porosity) of this landscape component in the entire landscape. This index has an important impact on biological protection and the distribution of matter and energy. The order of the patch density of each landscape component is NT > CKL > SL > ML > LKL > YT > CLKL > CZ > JJL > YNL > SY. The farmland has the largest patch density of 1.0 patch/km^2^, which means that the broad-leaved forest, the landscape matrix, is highly divided by farmland. The sum of the areas of the deciduous broad-leaved forest, evergreen broad-leaved forest, and evergreen–deciduous broad-leaved mixed forest, which have relatively large areas, accounts for more than 50% of the entire landscape area. And the patch density of the evergreen broad-leaved forest is greater than that of other broad-leaved forests, indicating that the evergreen broad-leaved forest plays a leading role among the broad-leaved forests. The patch density of the tung tree forest is similar to that of the deciduous broad-leaved forest, but the area of the tung tree forest is only half of that of the deciduous broad-leaved forest. It can be seen that the tung tree forest has a greater impact on the entire landscape than the deciduous broad-leaved forest.

### 3.4. Result of Landscape Fragmentation

Fragmentation is an important characteristic of landscape quality. The fragmentation index can quantify the degree of landscape fragmentation well. A larger value indicates a stronger degree of fragmentation and more severe interference. The fragmentation index (F) of the entire Nature Reserve is 0.9887, the patch fragmentation degree is 0.1732, the connectivity is 1.861, and the area-weighted shape index (AWS) is 425.3024. This indicates that the patches in the habitat of the Mrs Hume’s pheasant are relatively large in area and have a low degree of fragmentation. Therefore, the overall habitat in Jinzhongshan is suitable for the survival of the Mrs Hume’s pheasant. However, the connectivity of the patches is not high, and there are small-area patches that are not suitable for survival, resulting in a relatively high fragmentation index and a relatively high area-weighted shape index. The population of the Mrs Hume’s pheasant may still communicate through corridors or roads, etc., to reduce the impact of edge effects, thus forming an adaptation to fragmentation (Table 6).

Note: PLAND is the proportion of the landscape in the total area, NP is the number of patches, PD1 is the patch density of the landscape component, PD2 is the patch density of the entire landscape, LPI is the proportion of the largest patch in the landscape area, LSI is the landscape shape index, PAFRAC is the perimeter–area fractal dimension, and CONNECT is the connectivity.

### 3.5. Result of Landscape Diversity 

Fractal dimension (PAFRAC) can be intuitively understood as the non-integer dimension of irregular geometric shapes. This index is used to reveal the degree of boundary folding of each landscape component, and all landscape components follow the same fractal dimension rule. Since the number of farmland patches is less than 10, which has no practical significance, its fractal dimension is not calculated. The fractal dimensions of each landscape component do not differ much, indicating that the degrees of patch boundary folding are similar. The landscape component with the largest fractal dimension is the Pinus yunnanensis var. tenuifolia forest, followed by villages, while the masson pine forest has the smallest fractal dimension. This shows that the patch boundaries of the Pinus yunnanensis var. tenuifolia forest are the most tortuous and complex, while the fractal dimension indices of artificial forests such as Chinese fir forests, tung tree forests, and masson pine forests are relatively low because they are always reclaimed more neatly during artificial planting. The perimeter–area fractal dimension at the whole-landscape level is 1.337 (as shown in Table 5, Table 6 and Table 7), which is much less than 2, indicating that the degree of folding of the entire landscape boundary is low.

The landscape shape index reflects the complexity of landscape component patches. The larger the shape index, the more irregular and tortuous the patch shape is. The average shape index of Pinus yunnanensis var. tenuifolia forest patches, as the constructive species, is relatively high, indicating that its shape is complex. Among the shape indices of the entire forest landscape, the evergreen–deciduous broad-leaved forest has the largest value. The average shape indices of economic forests and evergreen broad-leaved forests are similar, but the area of economic forests is much smaller than that of evergreen broad-leaved forests, indicating that the evergreen broad-leaved forest landscape is more complex and its ecosystem is more stable.

The diversity index is a measure of the complexity and variability of various patches in the landscape. Generally, as the diversity index increases, the complexity of the landscape structural components also tends to increase. The landscape diversity index of Jinzhongshan is shown in Table 3, Table 4 and Table 5. From the perspective of the landscape interior, its Shannon’s evenness index (SHEI) is 0.845 (close to 1), indicating that the areas of each landscape component are relatively similar. The landscape contagion index (CONTAG) reflects the non-randomness or aggregation degree of different patch types in the landscape. If a landscape consists of many discrete small patches, its value is small; when the landscape is dominated by a few large patches or when patches of the same type are highly connected, its value is large. The landscape contagion value is 55.340, indicating that the proportions of different landscape types vary greatly, and the distribution of landscape components is uneven. In particular, the large differences between the three types of broad-leaved forests and farmland and villages result in a relatively large evenness index. Shannon’s diversity index (SHDI) is 2.03 (greater than 1), indicating that the landscape fragmentation in the study area is relatively serious (Table 7).

### 3.6. Result of Habitat Suitability Evaluation

Finally, based on the Sj values, habitats with Sj values greater than 0.333 are considered suitable habitats; those with Sj values between 0 and 0.333 are moderately suitable habitats; and those with Sj values equal to 0 are unsuitable habitats. By using the analytic hierarchy process, the overlay method, and geographic information system auxiliary software such as ArcView, it was determined that deciduous broad-leaved forests, deciduous–evergreen broad-leaved mixed forests, evergreen broad-leaved forests, and coniferous broad-leaved mixed forests are suitable habitats; coniferous forests and tung tree forests are moderately suitable habitats; and other woodlands are unsuitable habitats.

The area of Jinzhongshan is 38,716.605 hm^2^. The area of suitable habitats is 21,391.93 hm^2^, accounting for 55.25%; the area of moderately suitable habitats is 8160.343 hm^2^, accounting for 21.07%; and the area of unsuitable habitats is 9164.334 hm^2^, accounting for 23.67%. The area of the Jinzhongshan Nature Reserve is 20,924.4 hm^2^. The area of suitable habitats is 13,469.03 hm^2^, accounting for 64.31%; the area of moderately suitable habitats is 3521.10 hm^2^, accounting for 16.83%; and the area of unsuitable habitats is 3934.27 hm^2^, accounting for 18.81% (Table 8). From the results, it can be seen that after the establishment of the Guangxi Jinzhongshan National Nature Reserve, the proportion of habitats suitable for the Mrs Hume’s pheasant has increased significantly, rising from 76.32% in the Jinzhongshan area to 81.19% in the Jinzhongshan Nature Reserve.

**Figure 2:**
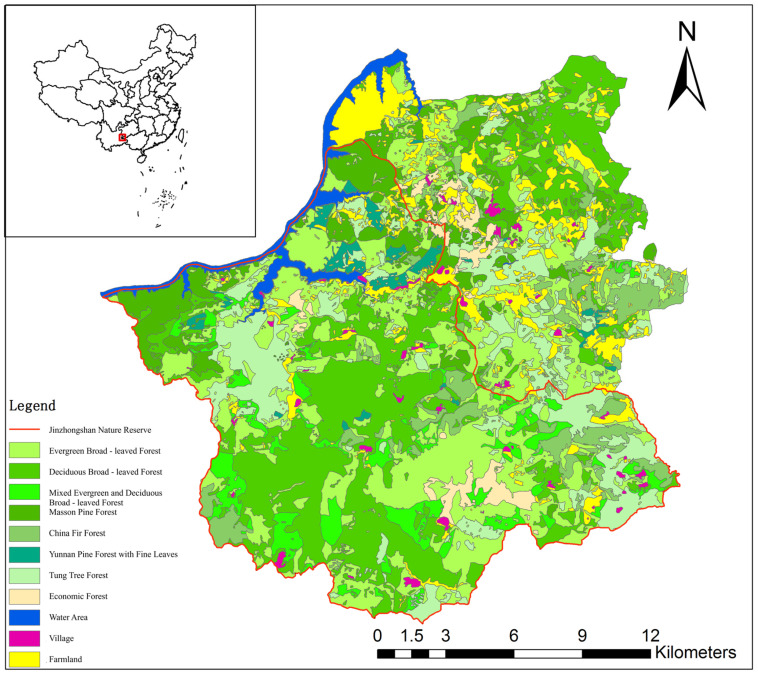
Mrs Hume’s pheasant habitat assessment in Jinzhongshan.

The habitat suitability pattern map of Hume’s pheasant in Jinzhongshan area (Figure 2) was drawn using the Geographic Information System (GIS 9.3)-assisted software ArcMap. Deciduous broad-leaved forests, mixed deciduous and evergreen broad-leaved forests, evergreen broad-leaved forests, and coniferous broad-leaved mixed forests are suitable habitats. Coniferous forests and tung tree forests are moderately suitable habitats. Water areas and other forested areas in blank spaces are unsuitable habitats. It can be seen from the map that the proportion of suitable habitats for Hume’s pheasant in the Jinzhongshan area is relatively large. However, it has already become fragmented, especially in areas outside the reserve, where the fragmentation is more severe, which has an impact on the survival of Hume’s pheasant.

## 4. Discussion

### 4.1. Analysis of Landscape Fragmentation 

Landscape pattern analysis is an important method in landscape ecology for analyzing landscape processes, and landscape indices are crucial indicators for landscape pattern analysis [2,3,4,7,12]. The landscape indices obtained in this study indicate that the landscape in Jinzhongshan is severely fragmented, especially in forest farms and collective forest areas outside the Nature Reserve. From the analysis results, it can be seen that at the whole-landscape scale, broad-leaved forests account for more than 50%, while the proportions of farmland and villages, which mainly affect landscape fragmentation, are less than 10%, and they do not affect the dominant position of broad-leaved forests. However, the patch density of villages in Jinzhongshan is the highest, and the number of farmland patches is the largest. Therefore, human interference may be the main cause of habitat landscape fragmentation. It was found that the connectivity of suitable habitats for the Mrs Hume’s pheasant is not high, indicating that the suitable habitats for the Mrs Hume’s pheasant in Jinzhongshan have been damaged to a certain extent. When using landscape pattern indices for analysis, most studies only consider the spatial scale and ignore the temporal scale [33]. Although traditional landscape indices can reflect the landscape configuration of macro-regions well, the local changes in landscape pattern indices in terms of space and type are different from the overall performance. There is a high correlation among landscape indices, which may lead to contradictions in the calculation results. As a result, the characteristics shown in the analysis of landscape processes cannot quantitatively reflect the impact process of human activities on landscape patterns, cannot accurately reveal the spatial information of the study area, and cannot represent the change rules of the landscape. This is also one of the reasons why landscape processes are not analyzed solely through landscape indices.

Habitat loss, habitat degradation, and poaching are the most significant threats to the Mrs Hume’s pheasant [8,22,27,44]. Habitat fragmentation causes the isolation of available resources and increases the edge effect, which in turn affects the lives of animals [50]. During the fragmentation process, there is a proportion threshold (10–50%) for suitable habitats in the landscape. Below this threshold, animal populations will decline rapidly due to the isolation effect. For birds with a wide range of activities, the lower limit of this threshold is even higher [7,12,36]. Although the proportion of suitable habitats in the Jinzhongshan area has not yet fallen below the minimum threshold, fragmentation has already become an obstacle to bird activities. If habitat fragmentation continues to intensify, it will pose a threat to the survival of the Mrs Hume’s pheasant. The number of Mrs Hume’s pheasant in the Jinzhongshan Nature Reserve exceeds 200 [47]; although the sample is limited, it is emphasized that this study provides the first fine-scale habitat fragmentation analysis for this species, serving as a baseline for future large-scale monitoring. A recent survey of pheasant populations in Jinzhongshan shows that Mrs Hume’s pheasant is also distributed in areas such as Malanqing outside the Nature Reserve, but it is difficult for populations in each patch to communicate, resulting in a certain degree of habitat fragmentation. The large distances between patches will affect the distribution of birds [17,32]. The habitat fragmentation index of the Mrs Hume’s pheasant in the Jinzhongshan Nature Reserve is 0.1732, and the fragmentation index is 0.9887, which is close to the maximum habitat fragmentation index of 0.9893 for the Elliot’s pheasant in northern Zhejiang, indicating that the habitats of the Mrs Hume’s pheasant in the Nature Reserve have been divided into multiple habitat fragments, and the degree of fragmentation is severe. There are collective forests around the Jinzhongshan Nature Reserve, where residents are engaged in forestry activities, and there are still residents inside the reserve who have not been relocated. Although logging and forest destruction are strictly prohibited, agricultural activities will definitely interfere with the activities of the Mrs Hume’s pheasant. More data are needed to support the formation mechanism of the habitat fragmentation of the Mrs Hume’s pheasant [44].

With the increase in population and the intensification of human interference, the habitats of the Mrs Hume’s pheasant in the Jinzhongshan area are constantly shrinking and being divided into fragmented patches [44,45]. As a result, the habitats of different populations of the Mrs Hume’s pheasant are still at risk of being isolated, and some populations are even facing the threat of habitat loss. At the same time, Mrs Hume’s pheasant has also been found in the Jinzhongshan Forest Farm and collective forests outside the Jinzhongshan Nature Reserve. In these areas, the traditional concept of slash-and-burn farming is deeply rooted, and deforestation for cultivation and illegal poaching still occur despite repeated prohibitions. Recently, in the newly discovered distribution points of the Mrs Hume’s pheasant in Guizhou Province, the habitats have also been threatened by fragmentation and are severely degraded [37,42]. In addition, fires and climate change also threaten the survival of the Mrs Hume’s pheasant in Thailand [14,17,18]. This study found that the selection coefficient for contiguous forest patches ≥ 50 ha in *Syrmaticus humiae* was 1.87 (*p* < 0.01), indicating that landscape fragmentation may significantly limit its dispersal ability. Combined with the trend of a 176% increase in the number of forest patches and a 43% decrease in average patch area in this region from 1990 to 2020 [27], it is inferred that the current habitat network may already affect gene flow among populations [44].

### 4.2. Analysis of Habitat Suitability Evaluation

At the landscape scale, the area, shape, and perimeter of patches affect species diversity, biological migration, and the spread of interior and edge species [2,7,12]. Through landscape analysis, it was found that there are large areas of broad-leaved forests with a low degree of fragmentation and patches of Pinus yunnanensis var. tenuifolia forest with good connectivity in the Jinzhongshan area, which are suitable for the Mrs Hume’s pheasant to inhabit. However, the Mrs Hume’s pheasant also chooses Chinese fir forests and tung tree forests with severe human interference and a high degree of fragmentation in the Ziyun County of Anshun City, Guizhou Province, China [46]. This is related to the excellent conditions in tung tree forests and Chinese fir forests, such as suitable water sources, good concealment, easy escape from natural enemies, and abundant food [42,46]. Since masson pine forests are used as economic timber, they are subject to particularly severe human interference. Although the connectivity of masson pine forests is relatively high, no activities of the Mrs Hume’s pheasant have been found, possibly because the interference factors exceed the tolerance range of the Mrs Hume’s pheasant.

The Habitat Suitability Index is a simple method for comprehensively evaluating habitat suitability based on the landscape scale. It uses spatial analysis methods to couple the habitat selection mechanism of species with landscape maps [7,8]. The advantage of this method is that it has relatively low requirements for the “occurrence point” data of the research object, and it is particularly suitable for species that are in urgent need of protection but lack systematic “occurrence point” data. At the same time, based on the habitat selection mechanism of the Mrs Hume’s pheasant [37,42], the rationality of the evaluation results is ensured to a certain extent, enabling the evaluation results to be applied in fields such as nature reserve management and the planning of key protected areas. However, this method also has drawbacks [47,48,49]. The weight values of various habitat-influencing factors are artificially set, which has a certain degree of subjectivity. Seasonal changes will affect the actual habitat use of the pheasant, and there may be a certain difference between the evaluation results and the actual habitats used by the Mrs Hume’s pheasant [49]. The study of animal life histories provides theoretical support for predicting and understanding landscape changes [12]. The seasonal changes in patch resources reflect the differences in resource requirements of animals at different life history stages, and a single patch often cannot meet the needs of the entire life history [38]. Especially during the breeding season, pheasants migrate between patches to find suitable breeding grounds. In terms of micro-habitat selection, early studies showed that the nest sites of the Mrs Hume’s pheasant are mainly selected at the base of trees in the forest, often covered by sparse grass and shrubs [17,32]. A study on the nest site selection of re-introduced Mrs Hume’s pheasant in Cenwanglaoshan Nature Reserve found that they can breed under Chinese fir forests [2,17,32]. However, no activities of the Mrs Hume’s pheasant under Chinese fir forests have been observed in Yunnan [12]. In the Jinzhongshan area, it was found that the Mrs Hume’s pheasant breeds under tung tree and Chinese fir forests. This indicates that due to the different availability of resources in different regions, there are differences in the nest site selection and habitat selection of the Mrs Hume’s pheasant [44,45]. Field surveys have found that during the breeding season, the Mrs Hume’s pheasant will move between different patches, sometimes even leaving the long-term inhabited patch for up to a month. The unevenness and singularity of patch resources force the Mrs Hume’s pheasant to spend more energy in searching for suitable resources [50,51,52].

### 4.3. Reasonable Conservation Measures

The top priority is to assess the current survival status of the Mrs Hume’s pheasant population, control the fragmented landscape that has emerged in the Jinzhongshan area, and strengthen the protection of the Mrs Hume’s pheasant through measures such as habitat restoration, corridor reconstruction, and enhancing public awareness of protection [44,53]. The following aspects can be considered to reduce the degree of landscape fragmentation:

(1) For different habitat types: For example, it is suggested to conduct light logging in Pinus yunnanensis forests once every 5 years to maintain a canopy density of 60–70%, so as to meet the foraging and hiding needs of the species;
(2) For fragmentation issues: It is suggested to construct ecological corridors dominated by evergreen broad-leaved trees between habitat patches with a distance of more than 500 m, and the width of the corridors should not be less than 30 m;
(3) For disturbance management: It is suggested to impose a ban on agricultural activities within 1 km of the core habitat during the breeding period (e.g., March–June), set up warning signs, and conduct regular inspections.

## 5. Conclusions

The Jinzhongshan region covers 38,716.6 hm^2^, including 1708 patches and 11 landscape types. Although the area of farmland and village only occupies 10%, their number and density have led Jinzhongshan habitats to fragment. The degree of connection of suitable habitat was found to be relatively low, and seven landscape indices were below 0.5, which implied that the extent of habitat fragmentation in Jinzhongshan for Mrs Hume’s pheasant is very high. The fragmentation index of Jinzhongshan Nature Reserve is 0.9887, landscape connectivity is 1.861, and AWS index is 425.3024. The broad-leaved forest, considered a matrix in the Jinzhongshan area, was the dominant landscape type controlling structure, function, and dynamic changes. The total suitable habitat of Mrs Hume’s pheasant (*Syrmaticus humiae*) was determined to be 29,552.3 hm^2^, accounting for 76.3% of the total study area; the suitable habitat of Mrs Hume’s pheasant in Jinzhongshan Nature Reserve was determined to be 16,990.1 hm^2^, accounting for 81.14% of the protected area. It is absolutely necessary and urgent to strengthen the management and protection of the Mrs Hume’s pheasant’s habitat to control its fragmentation. Therefore, we have provided some useful advice, such as habitat restoration and corridor reconstruction, which are beneficial to the conservation of Mrs Hume’s pheasant in this sensitive region.

## Figures and Tables

**Table 1:** Radio transmitter fixes of Mrs. Hume’s pheasant captured from Jinzhong Mountain, Guangxi.

Number	Sex	Capture Date	Radio Frequency (Hz)	Weight (g)	Location Number	Tracking Time (days)
1	♀	11 July 2011	150.482	564	31	34
2	♂	5 September 2011	150.152	608	27	16
3	♂	5 September 2011	150.133	796	11	19
4	♀	6 August 2012	150.100	617	12	12
5	♂	5 September 2011	150.060	939	9	12
6	♂	12 January 2012	150.080	912	11	25
7	♂	22 July 2012	150.318	886	5	5
8	♂	12 July 2012	150.666	909	5	5

**Table 2:** Definition and measurements of 20 variables involved in habitat selection.

	Variables	Unit	Plot Size	Method	Definitions and Measurement
1	Altitude	m	10 m × 10 m	GPS	This refers to the height of the site where the activity traces were detected, five classifications: 0–900, 901–1100, 1101–1300, 1301–1500, 1501–1750
2	Slope	°	10 m × 10 m	Horizontal ruler	This refers to the extent of calculated slope in degrees, three classifications: <20, 20–40, >40
3	Slope direction	°	10 m × 10 m	Compass	This refers to which direction and level the slope was facing, i.e., against or towards the sun, including sunny slope (S 67.5. E. S 22.5. W), half-sunny slope (N 22.5. E. S 67.5. E and S 22.5. W. N 67.5. W), and shady slope (N 67.5. W. N 22.5. E)
4	Slope position		10 m × 10 m	Estimation	This refers to the level of slanting of an angle on the surface of the mountain to the horizontal surface, three classifications: uphill, middle of the hill, downhill
5	Distance to habitat edge	m	10 m × 10 m	GPS	Five classifications, 0–100, 101–200, 201–300, 301–400, >400, based on the distance to habitat patch edge
6	Distance to water	m	10 m × 10 m	GPS	Four classifications, 0–200, 201–400, 401–600, >600, based on the distance from the center of quadrant to water
7	Distance to human resident	m	10 m × 10 m	GPS	Four classifications, 0–200, 201–400, 401–600, >600, based on the distance of the nearest resident
8	Distance to road	m	10 m × 10 m	GPS	Four classifications, 0–200, 201–400, 401–600, >600, based on the distance of the nearest road
9	Tree coverage	%	10 m × 10 m	Estimation	This refers to the coverage degree of tree crown, five classifications: 0–20, 21–40, 41–60, 61–80, >80
10	Shrub coverage	%	10 m × 10 m	Estimation	This refers to the coverage degree of shrubs, five classifications: 0–20, 21–40, 41–60, 61–80, >80
11	Herb coverage	%	10 m × 10 m	Estimation	This refers to the coverage degree of herbs, five classifications: 0–20, 21–40, 41–60, 61–80, >80
12	Leaf litter coverage	%	1 m × 1m	Estimation	This refers to the coverage degree of leaf litter, five classifications: 0–20, 21–40, 41–60, 61–80, >80
13	Height of tree	m	10 m × 10 m	Laser ranging device	This refers to the average height of trees, three classifications: <5, 5–10, >10
14	Density of tree		10 m × 10 m	Direct count	This refers to the number of trees, four classifications:0–5, 6–10, 11–20, >20
15	Height of shrub	m	10 m × 10 m	Laser ranging device	This refers to the average height of shrubs, three classifications: <1, 1–2, >2
16	Density of shrub		10 m × 10 m	Direct count	This refers to the number of shrubs, three classifications: <10, 11–30, >30
17	Height of herb species	cm	1 m × 1m	Direct count	This refers to the average height of herbs, four classifications: 0–20, 21–50, 51–100, >100
18	Density of herb		1 m × 1 m	Direct count	This refers to the number of herbs, three classifications: <10, 11–30, >30
19	Diameter of tree	cm	10 m × 10 m	Steel tape	The average diameter of the trees 1.3 m from the ground, three classifications: <10, 11–30, >30
20	Food abundance		1 m × 1 m	Sampler	Estimation, three classifications: sparse, average, rich; based on food availability of insect, including fruit, leaf, and herb

**Table 3:** Landscape classification of Mrs Hume’s pheasant in Jinzhongshan.

NO.	Type	Major Component
CKL	Evergreen broad-leaved forest	*Cyclobalanopsis glauca*, Castanopsis delavayi
LKL	Deciduous broad-leaved forest	Quercus acutissima, Quercus variabilis, Albizia kalkora
CLKL	Evergreen–deciduous broad-leaved mixed forest	Quercus acutissima, iquidambar formosana, Quercus variabilis
ML	Masson pine forest	Masson pine
SL	Chinese fir forest	Chinese fir forest
YNL	Pinus yunnanensis var. tenuifolia forest	Pinus yunnanensis var. tenuifolia
YT	Tung oil forest	Tung oil
JJL	Economic forest	*Castanea mollissima*, *Camellia oleifera*
SY	Water area	Rivers forming surface layers exceeding 5 m
CZ	Villages	Composed of multiple households
NT	Farmland	Corn sugarcane fields, etc.

**Table 4:** The criteria value for the habitat suitability assessment of Mrs Hume’s pheasant.

Influencing Factors		Most Suitable	Next Most Suitable	Less Suitable	Unsuitable
Tree	Height (m)	>15	10–15	5–10	<5
	Canopy density (%)	>60	40–60	20–40	<20
Shrub	Canopy density (%)	<20	20–40	40–60	>60
Grass	Height (m)	<0.6	0.6–0.8	0.8–1	>1
	Coverage (%)	<20	20–40	40–60	>60
Defoliation	Coverage (%)	>60	40–60	20–40	<20
	Thickness (cm)	>10	6–9	2–5	<1
Value (*Si*)		1	0.667	0.333	0

**Table 5:** Landscape pattern and properties of Mrs Hume’s pheasant in Jinzhongshan.

Type	Landscape Area (hm^2^)	Patch Average Area (hm^2^)	Gross Perimeter (m)	PLAND	NP	PD_1_	PD_2_	LPI	LSI	PAFRAC	CONNECT
Landscape	38,716.605	22.668	5,265,230	100	1708	62.69	4.412	25.606	34.4797	13.592	5.052
CKL	9727.48	31.379	1,332,910	25.125	310	3.187	0.801	7.082	33.778	1.317	0.357
LKL	9458.453	52.841	908,420	24.43	179	1.892	0.462	7.484	23.346	1.386	0.465
CLKL	2205.998	37.39	340,620	5.698	59	2.675	0.152	0.626	18.127	1.379	0.468
ML	3368.77	15.453	464,860	8.701	218	6.471	0.563	1.466	20.019	1.282	0.617
SL	2836.365	11.818	456,020	7.326	240	8.462	0.62	0.977	21.399	1.316	0.342
YNL	634.528	25.381	107,250	1.639	25	3.94	0.065	0.378	10.639	1.429	0.667
YT	4689.45	27.107	592,140	12.112	173	3.689	0.447	2.562	21.61	1.322	0.35
JJL	953.238	25.085	148,990	2.462	38	3.986	0.098	0.668	12.064	1.394	1.423
SY	1037.265	345.755	116,040	2.679	3	0.289	0.008	2.671	9.002	—	—
CZ	348.553	6.115	90,590	0.9	57	16.353	0.147	0.079	12.127	1.419	0.125
NT	3456.508	8.514	707,390	8.928	406	11.746	1.049	1.613	30.076	1.348	0.238

Note: PLAND refers to the proportion of the landscape in the total area; NP represents the number of patches; PD_1_ stands for the patch density of landscape components; PD_2_ denotes the patch density of the entire landscape; LPI is the proportion of the largest patch in the landscape area; LSI means the landscape shape index; PAFRAC is the perimeter–area fractal dimension; and CONNECT indicates the connectivity.

**Table 6:** Habitat fragmentation of Hume’s pheasant in Jinzhongshan Nature Reserve.

Total Area/km^2^	Patch Fragmentation	Index of Fragmentation (F)	Connectivity Index	AWS
209.242	0.1732	0.9887	1.861	425.3024

**Table 7:** Land metrics in Jinzhongshan.

Index	SHDI	SIDI	SHEI	PAFRAC	CONTAG (%)
Data	2.03	0.836	0.845	1.337	55.340

Note: SHDI stands for Shannon’s diversity index; SIDI represents Simpson’s diversity index; SHEI refers to Shannon’s evenness index; MSIEI denotes the modified Simpson’s evenness index; PAFRAC is the perimeter–area fractal dimension; and CONTAG indicates the landscape contagion value.

**Table 8:** Hume’s pheasant habitat assessment in Jinzhongshan and Jinzhongshan Nature Reserve.

Parameter	Suitable	Less Suitable	Unsuitable	Total
Jinzhongshan Region (hm^2^)	21,391.93	8160.343	9164.334	38,716.605
Percentage (%)	55.25	21.07	23.68	100
Jinzhongshan Nature Reserve (hm^2^)	13,469.03	3521.10	3934.27	20,924.4
Percentage (%)	64.31	16.83	18.86	100

## Data Availability

The data that support the findings of this study are not publicly available due to pending institutional data release approval but are available from the first author upon reasonable request. Interested researchers should contact Yuan Baodong at yuanbao365@163.com with a detailed research proposal to request access. All data requests will be reviewed to ensure compliance with the study’s ethical approval (approval code: GXJZS007]) and data protection regulations.
